# Consequences of warming and acidification for the temperate articulated coralline alga, *Calliarthron tuberculosum* (Florideophyceae, Rhodophyta)

**DOI:** 10.1111/jpy.13272

**Published:** 2022-07-02

**Authors:** Emily M. Donham, Scott L. Hamilton, Ivano Aiello, Nichole N. Price, Jennifer E. Smith

**Affiliations:** ^1^ University of California Santa Cruz Ecology and Evolutionary Biology 130 McAllister Way, Santa Cruz California 95060 USA; ^2^ Moss Landing Marine Laboratories San Jose State University 8272 Moss Landing Rd Moss Landing California 95039 USA; ^3^ Bigelow Laboratory for Ocean Sciences 60 Dr, East Boothbay Bigelow Maine 04544 USA; ^4^ Scripps Institution of Oceanography 9500 Gilman Dr, La Jolla California 92093 USA

**Keywords:** climate change, coralline algae, kelp forest, ocean acidification, photosynthesis, Rhodophyta, temperature

## Abstract

Global climate changes, such as warming and ocean acidification (OA), are likely to negatively impact calcifying marine taxa. Abundant and ecologically important coralline algae may be particularly susceptible to OA; however, multi‐stressor studies and those on articulated morphotypes are lacking. Here, we use field observations and laboratory experiments to elucidate the impacts of warming and acidification on growth, calcification, mineralogy, and photophysiology of the temperate articulated coralline alga, *Calliarthron tuberculosum.* We conducted a 4‐week fully factorial mesocosm experiment exposing individuals from a southern CA kelp forest to current and future temperature and pH/*p*CO_2_ conditions (+2°C, −0.5 pH units). Calcification was reduced under warming (70%) and further reduced by high *p*CO_2_ or high *p*CO_2_ x warming (~150%). Growth (change in linear extension and surface area) was reduced by warming (40% and 50%, respectively), high *p*CO_2_ (20% and 40%, respectively), and high *p*CO_2_ x warming (50% and 75%, respectively). The maximum photosynthetic rate (P_max_) increased by 100% under high *p*CO_2_ conditions, but we did not detect an effect of *p*CO_2_ or warming on photosynthetic efficiency (α). We also did not detect the effect of warming or *p*CO_2_ on mineralogy. However, variation in Mg incorporation in cell walls of different cell types (i.e., higher mol % Mg in cortical vs. medullary) was documented for the first time in this species. These results support findings from a growing body of literature suggesting that coralline algae are often more negatively impacted by warming than OA, with the potential for antagonistic effects when factors are combined.

**Abbreviations**
CCA: crustose coralline algae
OA: ocean acidification
*p*CO_2_
: partial pressure of CO_2_ gas
HgCl_2_
: mercuric chloride
PAR: photosynthetically active radiation

1

Changes in ocean chemistry due to climate change, such as ocean acidification (OA) and warming, are affecting marine organisms worldwide. The impacts of OA on marine organisms that build calcium carbonate shells and skeletons are generally negative (Kroeker et al. 2010, Cornwall et al. 2021), whereas the impacts of warming can be negative or positive depending on species‐specific thermal tolerances. While OA and warming often occur simultaneously in marine ecosystems, fewer studies have tested these multi‐stressor impacts in a fully factorial design. Yet, the combined effects of OA and warming are sometimes greater (or smaller) than would be predicted from either driver in isolation (Crain et al. 2008, Harvey et al. 2013) and even closely related taxa can differ greatly in the direction and magnitude of their responses to acidification and warming. Therefore, in order to more accurately predict the consequences of global change on species and ecosystems, it is necessary to further understand the mechanistic underpinnings of species responses to multiple environmental drivers (Kroeker et al. 2017).

Calcifying organisms are some of the main habitat‐forming species found in marine ecosystems. These ecologically important species are particularly susceptible to OA due to reductions in growth and calcification brought on by decreases in the saturation states of calcium carbonate minerals as a consequence of increases in dissolved CO_2_ (Doney et al. 2009). Calcifying taxa can, however, differ in the mineral composition of their skeletal material. Carbonate skeletons consisting of high Mg‐calcite (mol % Mg >4%) are more soluble than those made of low Mg‐calcite, aragonite, or calcite (Andersson et al. 2008). Some species have even been shown to change the polymorph of biominerals produced to decrease their solubility in acidified conditions (Ries et al. 2009). Furthermore, the mol % Mg of biominerals is positively correlated with temperature, suggesting that warming could increase biomineral solubility (Andersson et al. 2008). It is unknown, however, whether the relationship between mol % Mg and temperature is directly due to temperature or is a consequence of more rapid growth (Nash and Adey 2018). Furthermore, although increased incorporation of Mg into the crystal lattice of carbonate skeletons may make organisms more susceptible to OA, high Mg‐calcite has also been shown to improve elasticity and hardness properties of biominerals compared to pure calcite (Long et al. 2014), which could be crucial for species inhabiting high energy environments or at risk to grazing or predation. Importantly, organisms exhibiting mineralogical plasticity may be better prepared to deal with future change, if they are able to balance the benefits of Mg incorporation with the costs of increasing solubility in a more acidic and warmer environment.

Coralline red algae (Order Corallinales, Phylum Rhodophyta) are ecologically important calcifying macroalgae that precipitate high Mg‐calcite skeletons. They are ubiquitous members of coastal marine ecosystems around the world and provide structural support by acting as “cement” to bind benthic substrates or as free‐living aggregations (i.e., rhodolith or maërl beds; Riosmena‐Rodríguez et al. 2017). In addition, coralline algae provide refugia (articulated, branching, or free‐living forms) for many associated taxa and have been shown to facilitate recruitment by producing settlement cues and settlement substrate for both intertidal and subtidal marine communities (Steneck 1986, Williams et al. 2008, Siboni et al. 2020). Furthermore, calcification plays an important role in the growth and herbivore deterrence (McCoy and Kamenos 2018), preventing overgrowth and fouling by tissue sloughing (Pueschel et al. 2005), and protection from UV radiation (Gao and Zheng 2009). Due to their ecological importance on reefs and potential sensitivity to environmental change as calcifiers, coralline red algae have already become the focus of numerous climate change studies.

Responses of coralline algae to global climate change have largely been reported as negative (Hofmann and Bischof 2014, McCoy and Kamenos 2015, Cornwall et al. 2021) and most studies have focused on OA as the primary stressor (Cornwall 2019). Negative responses of coralline algae to OA include reduced recruitment (Kuffner et al. 2008), decreased calcification (McCoy and Kamenos 2015), decreased photosynthetic efficiency (Gao and Zheng 2009, Porzio et al. 2018), bleaching (Anthony et al. 2008), increased susceptibility to grazing (Johnson and Carpenter 2012, McCoy and Kamenos 2018), and weakening of the skeletal structure (Ragazzola et al. 2012). Studies that have assessed the impacts of warming on coralline algae suggest that the impacts of warming can cause bleaching (Huggett et al. 2018), decreased survival and growth of germlings (Page and Diaz‐Pulido 2020), reduced photosynthetic efficiency (Lei et al. 2020), reduced growth (Kram et al. 2015), reduced net calcification (Martin and Gattuso 2009), increased partial mortality (Diaz‐Pulido et al. 2012), and increased susceptibility to grazing (Johnson and Carpenter 2012). Although negative effects of OA or warming on coralline performance are most common, there are also examples where corallines have been shown to be highly resilient or resistant to acidification or warming, suggesting responses are species–specific and/or context dependent (Cornwall 2019). Furthermore, fewer studies have assessed how concurrent warming and OA will impact coralline algae compared to studies testing the effects of either driver in isolation. Those that have tested multiple stressors suggest that warming has the potential to both further exacerbate (Martin and Gattuso 2009, Diaz‐Pulido et al. 2012, Rasher et al. 2020) or ameliorate the impacts of OA (Johnson and Carpenter 2012, Williams et al. 2014), depending on taxa. Importantly, since environmental drivers are often changing simultaneously, it is crucial to examine both the independent and interactive effects of OA and warming on species responses.

Despite the increasing number of studies assessing the impacts of climate change on coralline red algae, the majority of these studies are conducted on encrusting morphotypes (Cornwall 2019). Understudied articulated morphotypes, however, may be especially important as they provide habitat for diverse macrofaunal assemblages within their complex 3‐D structure (McLean 1962, Kelaher and Castilla 2005). *Calliarthron tuberculosum* is a subtidal and intertidal temperate articulated coralline alga found along the west coast of North America from at least as far south as Baja, Mexico, to as far north as British Columbia, Canada (Gabrielson et al. 2011). *Calliarthron tuberculosum* inhabits the California Current System (CCS) which is characterized by naturally dynamic oceanographic conditions driven by seasonal upwelling (Bakun et al. 2015). The effects of climate change on oceanographic conditions within the CCS are forecasted to change especially rapidly (i.e., acidification and deoxygenation; Cheresh and Fiechter 2020) and it is unclear whether marine species living within these dynamic environments will be able to cope with the unprecedented rate of change, though coralline algae from variable intertidal ecosystems have shown some resilience to environmental stressors associated with climate change (Noisette et al. 2013, Egilsdottir et al. 2016, McCoy and Widdicombe 2019). To gain a more comprehensive understanding of the consequences of climate change on coralline algae and their ecosystems, it is important to study other articulated species that have adapted/acclimated to naturally dynamic ecosystems, such as subtidal kelp forests.

The overarching goal of our study was to improve our understanding of the impacts of OA and warming on coralline red algae by addressing the following question: How will OA and warming affect the growth, calcification, and mineralogy of the abundant temperate articulated coralline red alga, *Calliarthron tuberculosum*? We hypothesized that 1) OA and warming will decrease growth, calcification, and photosynthetic efficiency; and 2) OA will decrease the mol % Mg incorporated into biominerals, but warming will increase the mol % Mg.

## 2 MATERIALS AND METHODS

### 2.1 Study site and collections

The articulated coralline red alga, *Calliarthron tuberculosum,* was selected as our study organism due to its ecological importance, wide spatial distribution, and rapid growth rates (up to 1.8 cm^2^ · month^−1^; Martone 2010). We collected *C. tuberculosum* subtidally (~13 m depth) using SCUBA on August 8, 2013 at Mia's Reef in San Diego, California (32°51′14.8“ N, 117°16’52.4” W). We gently dislodged *C. tuberculosum* individuals from the substrate keeping the holdfasts intact, placed them in collection bags, and then transported them to the experimental aquarium at Scripps Institution of Oceanography (SIO) in coolers filled with seawater. *Calliarthron tuberculosum* individuals were identified in the field morphologically and then confirmed in the laboratory based on caliper measurements of apical intergenicula (Gabrielson et al. 2011).

pH, temperature, and irradiance measurements were recorded at our collection site to provide an environmental context and aid in the interpretation of our experimental results. Bottom irradiance data were collected every 15 min from October 6, 2013 to October 25, 2013 using an LI‐193 spherical sensor (Biospherical Instruments Inc.). We deployed an autonomous pH and temperature logger, SeaFET (Martz et al. 2010), at Mia's Reef from November 12, 2012 to April 12, 2014. The SeaFET recorded pH and temperature every 15 min and was used to understand natural diurnal and tidal fluctuations in pH and temperature. We collected discrete water samples for carbonate chemistry directly next to the SeaFET, using a custom‐made, diver deployable Niskin bottle, approximately once a month to calibrate pH measurements. At the surface, we transferred water samples to 500‐mL Corning brand Pyrex sample bottles and immediately spiked samples with 240 μL of HgCl_2_ solution. We measured pH with a spectrophotometer (Shimadzu, UV‐1800) and total alkalinity using open‐cell titration on triplicate samples (Metrohm, 905 Titrando) following standard protocols (Dickson et al. 2007). We calculated salinity from seawater density using a densiometer (Mettler Toledo, DX45). We calculated in situ pH using spectrophotometric pH, total alkalinity, salinity, and in situ temperature and pressure as inputs to the program CO2SYS (Pierrot et al. 2006) with stoichiometric dissociation constants defined by Mehrbach et al. (1973) and refit by Dickson and Millero (1987). We conducted SeaFET calibrations using guidelines outlined in Bresnahan et al. (2014).

### 2.2 Seawater bubbling system for ocean acidification and warming experiments

After collection, we transported individuals to the laboratory at SIO and held samples in flow‐through seawater under full‐spectrum 54 W Giesemann T‐5 fluorescent bulbs that approximated ambient light conditions in the field, for 1 month prior to the start of the experiment. In order to simulate the possible impacts of OA and warming on near‐shore ecosystems, we used a seawater bubbling system to manipulate *p*CO_2_ and temperature (described in Kram et al. 2015). Briefly, the seawater system bubbled an air or CO_2_‐air gas blend using a manifold with individual air stones supplied to individual 1‐liter glass mason jars (mesocosms) supplied with flow‐through seawater. After a 1‐month acclimation to ambient laboratory conditions, we placed individual specimens of *Calliarthron tuberculosum* (~1 g wet weight) directly into one of four treatment levels (*n* = 7 per treatment): (1) ambient temperature, ambient *p*CO_2_, (2) high temperature, ambient *p*CO_2_, (3) ambient temperature, high *p*CO_2_, and (4) high temperature, high *p*CO_2_ for 28 d. To create high *p*CO_2_ conditions that simulate future ocean chemistry, we lowered seawater pH by 0.5 ± 0.05 units (IPCC 2013) on top of the natural variability present in the incoming seawater by continuously bubbling a CO_2_‐air gas blend into individual mesocosms. To create ambient *p*CO_2_ conditions, we supplied ambient mesocosms with air originating from the same source as the CO_2_‐air gas blend used to obtain the high *p*CO_2_ treatment conditions. We maintained half of the mesocosms at ambient temperature and half of the mesocosms at +2°C above ambient to simulate levels of warming predicted by the year 2100 (IPCC 2013). Each temperature treatment was maintained in three replicate water baths using submersible aquarium heaters. Since our sample size was *n =* 7 per treatment, 2–3 replicate 1‐liter mason jars per treatment were placed in each water bath (*n* = 1 individual per jar). Our photoperiod was a 12:12 day:night cycle for the entirety of the experiment. We used a PAR sensor (QSL‐2200, Biospherical Instruments Inc.) to measure irradiance once weekly in all experimental jars.

To monitor experimental conditions, we collected temperature and pH data daily at midday (13:00 PST ± 2 h) in all aquaria using a HACH HQ40d handheld glass electrode pH probe calibrated each day with certified Tris buffer from the laboratory of Dr. Andrew Dickson at SIO. If the experimental pH drifted above or below the desired 0.5 ± 0.05 units below ambient, we made minor adjustments to bubbling rates in individual aquaria. To monitor carbonate chemistry parameters in the experiment, we collected discrete water samples in 500‐mL Corning brand Pyrex sample bottles from three control jars (without samples) and two randomly assigned specimen jars from each treatment at the beginning, middle, and end of the experiment. Only a subset of jars was sampled due to logistical constraints. We measured total dissolved inorganic carbon (DIC_T_) using a Single Operator Multi‐parameter Metabolic Analyzer (SOMMA) in the laboratory of Dr. Andrew Dickson (SIO). We measured total alkalinity (A_T_) via open‐cell acid titration using a Metrohm Dosimat Model 665 and Thermo Scientific Ross potentiometric pH probe and meter. We determined salinity using a densitometer (Mettler Toledo DE45) and calculated carbon species and saturation state based on measured DIC_T_, A_T_, and salinity using CO2SYS and stoichiometric dissociation constants defined by Mehrbach et al. (1973) and refit by Dickson and Millero (1987).

### 2.3 Net calcification

To measure net calcification rates, we first cleaned all specimens of epiphytes using forceps and a soft‐bristled brush and then weighed individuals using the buoyant weight method at the beginning and end of the experiment. We placed samples in a metal basket completely submerged in seawater. We attached the metal basket to the monofilament line and suspended it from a weigh‐below balance. The buoyant weight method was used since it is accurate at measuring the weight of calcium carbonate in calcified algae and does not take into account the weight of fleshy material, which is similar in density to seawater (Davies 1989). Percent change in calcified weight was calculated as, $\frac{W_{f}-W_{i}}{W_{f}}$, where *W*_*i*_ is the initial weight and *W*_*f*_ is the final weight.

### 2.4 Growth

Twenty‐four h before the start of the experiment, we placed samples in a 1‐L beaker with 0.02% Calcofluor White (Fluorescent Brightener 28, Sigma‐Aldrich) in seawater for 5 min in order to stain growing tips before skeletal material was deposited in response to the treatment conditions (Martone 2010). To measure growth, we calculated the linear extension and surface area of new growth deposited after the calcofluor stain. In order to visualize the calcofluor stain, we examined tips using a dissecting scope illuminated with an ultraviolet lamp (315–400 nm). We then randomly selected and removed stained tips at the closest geniculum behind the leading edge of the stain using forceps. We took photographs of three replicate tips in Image‐Pro Plus Software (Media Cybernetics, Inc.) using a Leica microsystems camera fitted to a dissecting scope (Leica MZ 12_5_, Leica Biosystems). We then used ImageJ software to calibrate the field of view using an objective micrometer (0–1 mm/100) and measure the length from the apex of the stained region to the tip of the branch to obtain a linear extension. From these same photographs, the planar area was also calculated within ImageJ as the surface area of new growth after the calcofluor band. We pooled all three tip measurements per individual for subsequent statistical analyses.

### 2.5 Photophysiology

In order to compare differences in photosynthetic efficiency, we calculated photophysiological parameters experimentally. We randomly selected samples (*n* = 3) from each treatment level and placed each individual into a separate 1.5‐L polycarbonate incubation container covered with a polycarbonate lid with an airtight rubber seal. To control for changes in dissolved oxygen concentrations due to water column processes, we used one container with no sample as a control. We obtained *p*CO_2_ conditions at the beginning of the incubations by bubbling either air or the same CO_2_‐air gas blend used during the experiment into a 20‐L carboy until the desired pH was obtained. We incubated containers in the light (irradiances: 19, 36, 60, 103, 178, 198, 344, 392, and 500 μmol photons · m^−2^ · s^−1^) and dark (0 μmol photons · m^−2^ · s^−1^) for 45 min. We measured light levels within each chamber by submerging the wand of the PAR sensor (QSL‐2200, Biospherical Instruments Inc.) and then covering with the polycarbonate lid. In between each light step, we opened the incubation containers and seawater was fully replenished. To maintain constant treatment temperatures, we submerged containers in a water bath. We used magnetic stir bars to stir seawater within each container and disrupt diffusive boundary layers. Occasionally stir bars did not function properly and were flagged during the incubations. As a result, 11 light steps were removed across all individuals due to improper mixing; however, no fewer than six light steps were used in any individual model. HACH 40D dissolved oxygen (DO) probes were inserted into each chamber via a gas‐tight port and measured DO concentrations within sealed chambers every minute. All DO probes were calibrated using a single point 100% saturation calibration. We calculated net production (NP, μmol O_2_ · g^−1^ · min^−1^) by plotting DO versus time at time t_0 + 10_ to t_0 + 20_, where t_0_ indicates the sealing of the incubation chamber. *NP* is the slope of the local linear regression fit at each light level using LoLinR in R (Olito et al. 2017). To calculate photophysiological parameters, *NP* versus irradiance data were fit to the equation:

(1) $$\mathrm{NP}=P_{\max}\times \left(1-e^{\frac{-\alpha *I}{P_{\max}}}\times e^{\frac{-\beta *x}{P_{\max}}}\right)$$

where *P*_*max*_ is the maximum photosynthetic rate, α is the photosynthetic efficiency, and β is the irradiance at which photoinhibition begins (Jassby and Platt 1976) using nls in R (stats package; R Studio Team 2019).

### 2.6 Mineralogy

A Scanning Electron Microscope (SEM; 3400n, Hitachi) with an attached Energy Dispersive X‐ray Spectrometer (EDS; Energy 250, INCA) was used to examine the spatial variability in mineralogy, and specifically changes to the mol % Mg incorporated into biominerals as a consequence of warming and acidification. Using EDS, Mg^2+^, and Ca^2+^ concentrations were measured in the calcified cell walls surrounding both medullary and cortical cells. Immediately following final calcification measurements (buoyant weighing), samples were placed in a drying oven at 60°C for 48 h. Each sample was visualized under a dissecting microscope with UV light projected onto the sample to visualize the calcofluor white stain. Using forceps and a razor blade, thallus material deposited after the fluorescent dye marker was removed from the tips of two different branches and placed aside for subsequent SEM and EDS analysis. Each subsample was secured to an individual SEM stub with resin (EPO‐TEK), polished, and gold coated. We quantified differences in the composition of Mg^2+^ and Ca^2+^ incorporated into biogenic carbonates by conducting spot analysis with the EDS at nine points underlying a 3x3 grid matrix. At each spot, we recorded the identity of the cell type (cortical versus medullary). We conducted these analyses on two separate branch tips for each individual. An accelerating voltage of 15 kV and emission current intensity of 60 mA were used for EDS analyses. To reduce error in measurements of Mg^2+^ and Ca^2+^ due to variability in the interaction volume of spot analysis (Nash and Adey 2017), only spot analyses with a Wt % (relative concentration of all elements in mass) equal to 80–120 were considered. The mol % Mg at each spot analysis was calculated as

(2) $$\frac{\mathrm{At}\%\mathrm{Mg}}{\mathrm{At}\%\mathrm{Mg}+\mathrm{At}\%\mathrm{Ca}}\times 100$$

where At%Mg and At%Ca are determined from EDS. For each individual, mol % Mg from three spot analyses for each cell type, corresponding to those closest to a Wt % = 100, was pooled for subsequent statistical analyses.

### 2.7 Statistical Methods

Calcification rates, linear extension, surface area, and mol % Mg were compared between treatments using two‐way factorial ANOVAs with fixed factors of *p*CO_2_ and temperature. Shapiro–Wilk tests (W) were calculated on the residuals to test for normality and homogeneity of variances. In the case where a response variable did not meet the assumptions of normality, it was square root transformed and analyses were rerun on transformed data. A two‐sample t‐test was used to compare differences between mol % Mg of cortical and medullary cells pooled across treatments. A two‐way ANOVA, with fixed factors of *p*CO_2_ and temperature, was run on photophysiological parameters *P*_max_ and α. All statistical analyses and model fitting were conducted in R (RStudio Team 2019).

## 3 RESULTS

### 3.1 Study site conditions

**Fig. 1:**
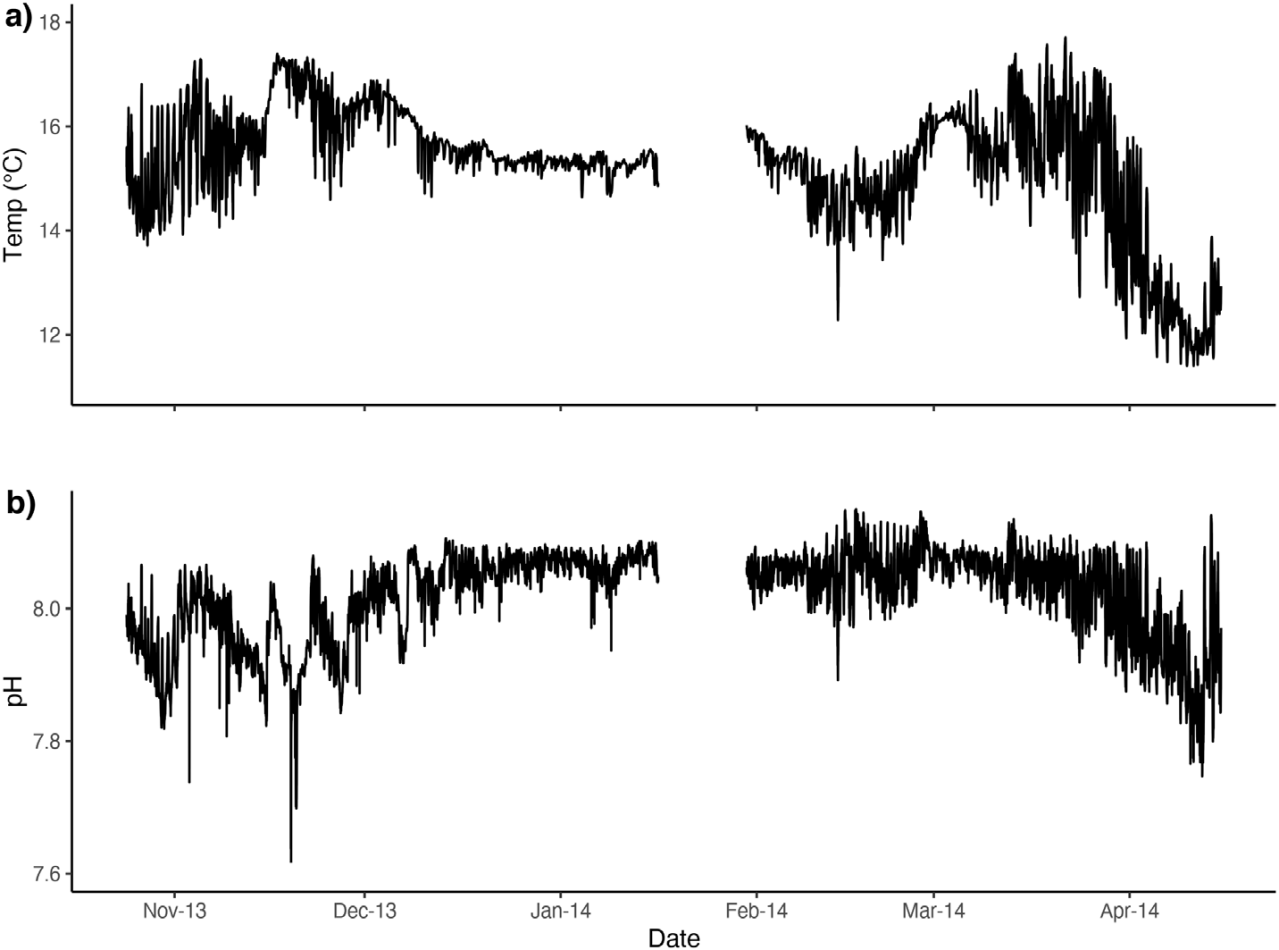
Time series of in situ (a) temperature (°C) and (b) pH_sw_ collected from autonomous SeaFET sensor deployed at Mia's Reef, San Diego, CA. The instrument was deployed on the benthos within a kelp forest at 15 m depth and data were collected every 15 min.

The 1st year of SeaFET logger measurements (November 2012–October 2013) was not usable due to biofouling and equipment malfunction. From October 23, 2013 to April 12, 2014 in situ measurements of pH and temperature from the autonomous SeaFET logger recorded a range in pH_sw_ from 7.57 to 8.15 with a mean pH_sw_ = 8.02 and a range in temperature from 11.40 to 17.71°C with a mean = 15.28°C (Fig. 1). The mean peak daily irradiance at ~13 m depth was ~65 μmol photons · m^−2^ · s^−1^ from October 6, 2013 to October 25, 2013 (Donham et al. 2021).

### 3.2 Experimental conditions

Four distinct *p*CO_2_ x temperature treatments were maintained for the duration of the 28‐d experiment (Table 1). Ambient *p*CO_2_ treatments were ~ 0.50 pH units higher and ~ 1200 μatm lower than the high *p*CO_2_ treatments. High‐temperature treatments were 2°C higher than ambient temperature treatments. The mean peak irradiance levels in mesocosms were 23 μmol photons · m^−2^ · s^−1^ (Table 1).

**Table 1:** Average environmental conditions in the field at Mia's Reef, San Diego, CA (collection site) from SeaFET (*temperature and pH*) and PAR sensor (*irradiance*), and mean seawater chemistry for experimental mesocosms obtained at the beginning, middle, and end of the 28‐day experiment (± SE, *except Field Temp and pH which are ± SD*). Discrete samples from mesocosms both with and without macroalgae were pooled within treatments.

Source of Variation	Temp	Salinity	Mean Peak Irradiance (μmol photons · m^−2^ · s^−1^)	pH_SW_	*p*CO_2_ (μatm)	A_T_ (μmol · kg^−1^)	DIC_T_ (μmol · kg^−1^)	HCO_3_^−^ (μmol · kg^−1^)	CO_3_^2−^ (μmol · kg^−1^)	CO_2_ (μmol · kg^−1^)	Ω _Calcite_	Ω _Aragonite_
Field	15.28 ± 1.17	‐	64.98 ± 9.70	8.02 ± 0.07	‐	‐	‐	‐	‐	‐	‐	‐
Amb *p*CO_2_, Amb temp	13.53 ± 0.03	33.49 ± 0.02	25.61 ± 1.66	7.96 ± 0.00	482 ± 4	2104 ± 7.11	2064 ± 7.09	1924 ± 6.58	121 ± 1.09	19.0 ± 0.16	2.9 ± 0.03	1.9 ± 0.02
Amb *p*CO_2_, High temp	15.43 ± 0.33	33.59 ± 0.04	24.68 ± 2.34	7.95 ± 0.01	493 ± 11	2109 ± 4.55	2066 ± 3.81	1919 ± 2.88	129 ± 1.62	18.3 ± 0.26	3.1 ± 0.04	2.0 ± 0.02
High *p*CO_2_, Amb temp	13.57 ± 0.18	33.48 ± 0.07	25.96 ± 1.80	7.42 ± 0.06	1874 ± 237	2187 ± 6.83	2241 ± 7.02	2128 ± 3.43	39 ± 5.04	73.7 ± 9.27	0.9 ± 0.12	0.6 ± 0.08
High *p*CO_2_, High temp	15.78 ± 0.19	33.61 ± 0.05	21.11 ± 0.86	7.45 ± 0.05	1802 ± 240	2190 ± 5.84	2235 ± 14.35	2122 ± 10.67	46 ± 5.06	66.1 ± 8.63	1.1 ± 0.12	0.7 ± 0.08

### 3.3 Net calcification

**Fig. 2:**
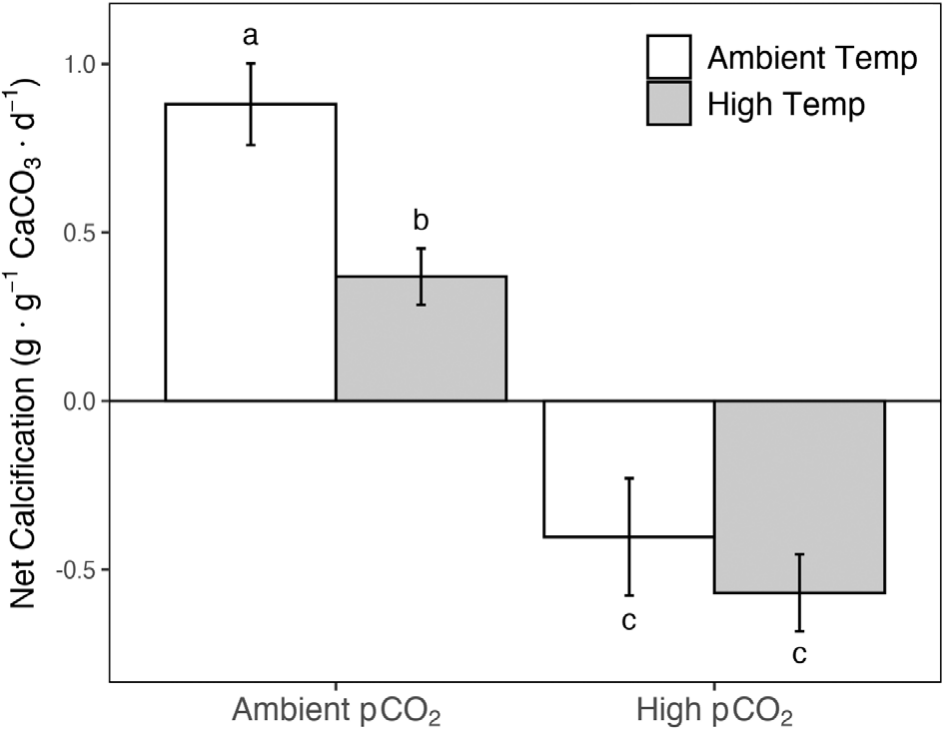
Percent change in buoyant weight of *Calliarthron tuberculosum* in the four factorial *p*CO_2_ and temperature treatments. Shared letters above or below error bars indicate mean change in buoyant weight did not differ between treatments. Error bars denote ± SE.

We observed a substantial reduction in buoyant weight of *Calliarthron tuberculosum* in the high *p*CO_2_ treatments over the course of the experiment, regardless of the temperature (Fig. 2). Buoyant weight was also negatively affected by increasing temperature, but there was no effect on the interaction (*p*CO_2_ *F*_1,24_ = 8.643, *P* = <0.0001; Temp *F*_1,24_ = 7.074, *P* = 0.014; *p*CO_2_*Temp *F*_1,24_ = 1.840, *P* = 0.188; Fig. 2). Under ambient *p*CO_2_ conditions, increased temperature reduced calcification rates (i.e., change in buoyant weight) by roughly 70%, but still resulted in the net positive growth of individuals. In contrast, high *p*CO_2_ treatments resulted in the net dissolution of CaCO_3_ over the 28‐d experiment, with no difference in dissolution rates between individuals reared in either temperature treatment. This corresponds to a ~ 150% reduction in CaCO_3_ in high *p*CO_2_ conditions in either temperature treatment compared to ambient *p*CO_2_ and ambient temperature conditions.

### 3.4 Growth

**Fig. 3:**
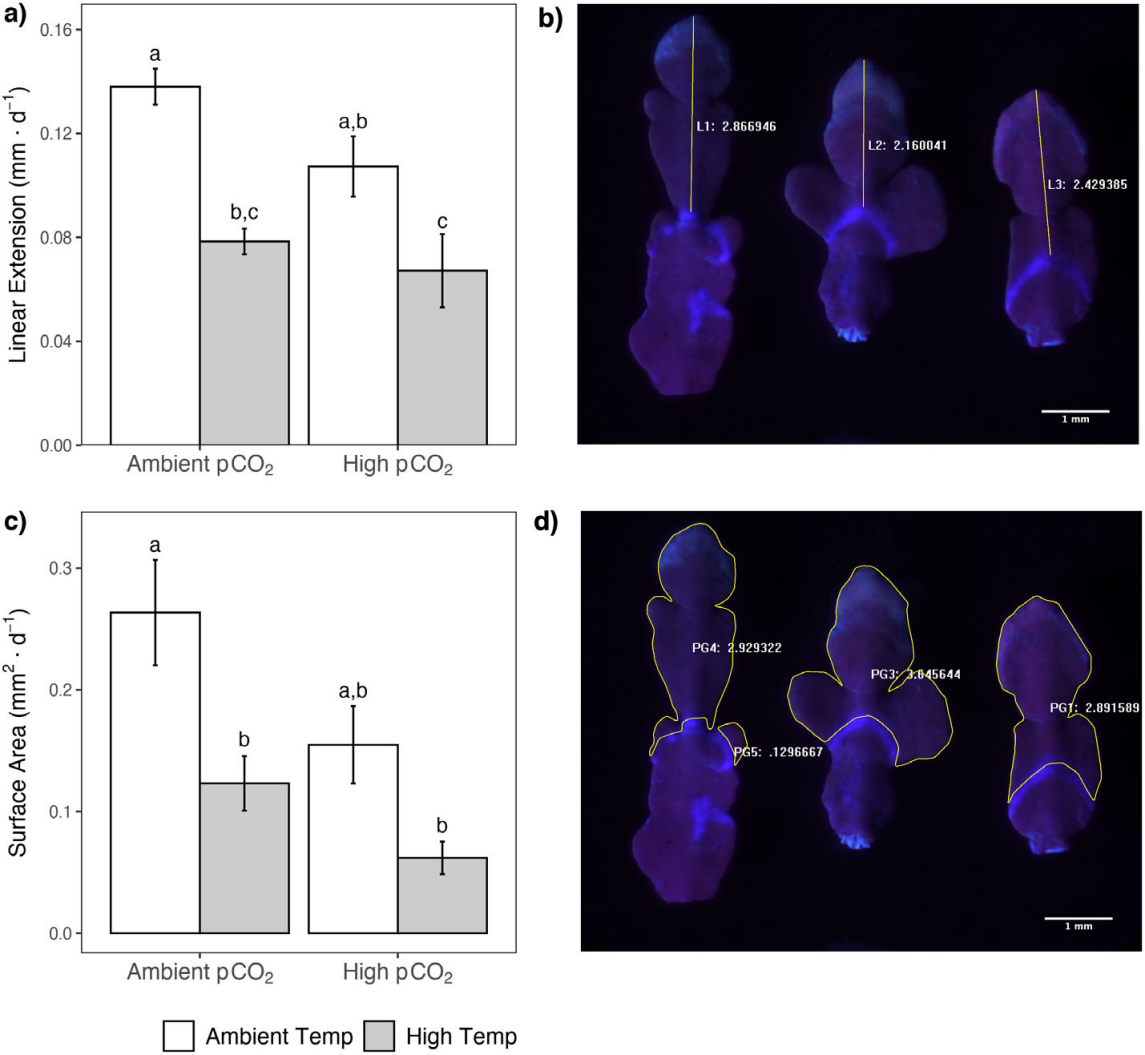
Growth rates as (a) linear extension and (c) surface area for *Calliarthron tuberculosum* in the four factorial *p*CO_2_ and temperature treatments. The shared letters above error bars indicate mean growth rate did not differ between treatments. Error bars denote ± SE. In panels (b and d), the Calcofluor stained region is depicted as a band on three growing tips from *C. tuberculosum*. Line (b) and outline (d) show linear extension or surface area measurements of new growth material during experimental rearing. [Color figure can be viewed at wileyonlinelibrary.com]

We observed that elevated temperature and high *p*CO_2_ decreased linear extension of *Calliarthron tuberculosum* branches (Fig. 3, a and b), but there was no *p*CO_2_ x temperature interaction (*p*CO_2_ *F*_1,24_ = 4.335, *P* = 0.048; Temp *F*_1,24_ = 24.463, *P* = <0.0001; *p*CO_2_*Temp *F*_1,24_ = 0.932, *P* = 0.344). Elevated temperature decreased linear extension by roughly 40% in ambient *p*CO_2_ conditions. Under high temperature and high *p*CO_2_, the linear extension was further reduced by 50%. However, under ambient temperature and high *p*CO_2_, the linear extension was only reduced by 20% compared to ambient temperature and ambient *p*CO_2_. We also found decreases in the surface area of new growth of branch tips (Fig. 3, c and d) in the elevated temperature and high *p*CO_2_ treatments, but no *p*CO_2_ x temperature interaction (*p*CO_2_ *F*_1,24_ = 8.100, *P* = 0.009; Temp *F*_1,24_ = 15.262, *P* = 0.0007; *p*CO_2_*Temp *F*_1,24_ = 0.631, *P* = 0.435). Increased temperature decreased surface area by ~50% in ambient *p*CO_2_ conditions compared to controls. Under high temperature and high *p*CO_2_, the surface area was reduced by 75%. Under ambient temperature and high *p*CO_2_, the surface area declined by 40%.

### 3.5 Photophysiology

**Fig. 4:**
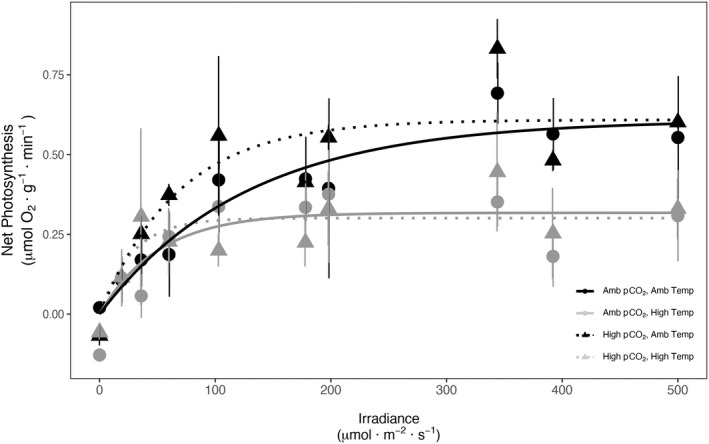
Net photosynthesis versus irradiance curves for *Calliarthron tuberculosum* after rearing in four factorial *p*CO_2_ and temperature treatments for 28 d. Net photosynthesis was expressed in terms of oxygen production.

Since there was little evidence of photoinhibition, models were fit with a beta set to zero (Fig. 4), and therefore, this parameter was not included in any further analyses. There were no effects of *p*CO_2_, temperature, or the *p*CO_2_ x temperature interaction on the photosynthetic efficiency, α (*p*CO_2_ *F*_1,8_ = 1.319, *P* = 0.284; Temp *F*_1,8_ = 0.295, *P* = 0.602; *p*CO_2_*Temp *F*_1,8_ = 0.152, *P* = 0.707; Table 2, Fig. 4). We did, however, find an effect of temperature on the maximum photosynthetic rate P_max_, with a roughly 50% decrease in this parameter under high‐temperature conditions (Table 2) but no effects of *p*CO_2_ or the *p*CO_2_ x temperature interaction (*p*CO_2_ *F*_1,8_ = 0.002, *P* = 0.963; Temp *F*_1,8_ = 16.363, *P* = 0.004; *p*CO_2_*Temp *F*_1,8_ = 0.161, *P* = 0.699; Fig. 4).

**Table 2:** Mean photophysiological parameters of *Calliarthron tuberculosum* reared under different treatments of *p*CO_2_ (ambient and high) and temperature (ambient and high). *N* = 3 individuals per treatment.

Treatment	*P*_max_ (± SE)	*α* (± SE)
Amb *p*CO_2_, Amb temp	0.690 ± 0.037	0.005 ± 0.003
Amb *p*CO_2_, High temp	0.319 ± 0.106	0.006 ± 0.002
High *p*CO_2_, Amb temp	0.652 ± 0.056	0.010 ± 0.003
High *p*CO_2_, High temp	0.348 ± 0.110	0.016 ± 0.011

### 3.6 Mineralogy

**Fig. 5:**
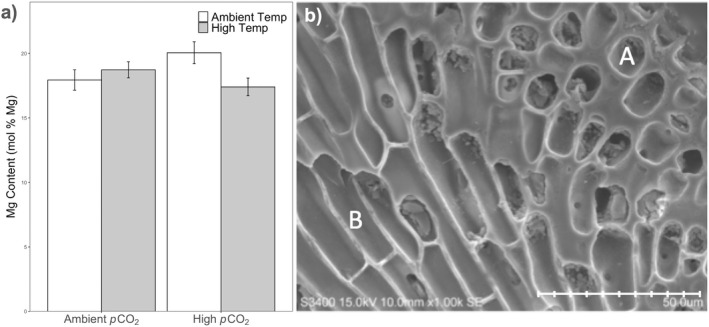
(a) Mol % Mg found in medullary cell walls of *Calliarthron tuberculosum* after rearing in four factorial *p*CO_2_ and temperature treatments for 28 d. Error bars denote ± SE. (b) Scanning electron microscopy (SEM) image at 1000x. Image depicts circular cortical cells (A) and elongated internal medullary cells (B). Scale bar = 50 μm.

We did not detect an effect of *p*CO_2_, temperature, or their interaction on the mol % Mg incorporation into cortical cells (*p*CO_2_ *F*_1,24_ = 0.306, *P* = 0.586; Temp *F*_1,24_ = 0.621, *P* = 0.439; *p*CO_2_*Temp *F*_1,24_ = 0.517, *P* = 0.479; Fig. 5). We did, however, find an interaction between *p*CO_2_ and temperature in medullary cells where increased *p*CO_2_ increased the mol % Mg at ambient temperature but decreased the mol% Mg at high temperature (*p*CO_2_ *F*_1,24_ = 0.285, *P* = 0.599; Temp *F*_1,24_ = 1.572, *P* = 0.222; *p*CO_2_*Temp *F*_1,24_ = 5.366, *P* = 0.029; Fig. 5). Further analysis comparing differences between cell types showed lower mol % Mg in medullary cells (mean ± SEM = 18.5 ± 0.4) versus cortical cells (mean ± SEM = 14.8 ± 0.3; *t*‐test: *t*_*54*_ = 7.167, *P* = <0.0001).

## 4 DISCUSSION

Work on temperate, tropical, and subtropical reefs has shown detrimental impacts on numerous physiological traits in calcareous biota exposed to high *p*CO_2_ and elevated temperature conditions (Kroeker et al. 2013). This study assessed the physiological and ecological responses of an important habitat forming articulated coralline alga, *Calliarthron tuberculosum,* to short‐term (28 d) OA and warming in a factorial design. We found that both ocean acidification and warming reduced growth and net calcification, yet had few impacts on photophysiology. The exception was a significant decrease in the maximum photosynthetic rate (P_max_) in response to increased temperature. We did not find any significant main effects of acidification or warming on mol % Mg of biominerals; however, we did find a significant interaction between OA and warming within medullary cells, such that high *p*CO_2_ increased the mol % Mg at ambient temperature but decreased the mol % Mg at elevated temperature. We also found that medullary cells have lower mol % Mg than cortical cells. Together these results suggest that the impacts of OA and warming on *C. tuberculosum* physiology and mineralogy are complex but are likely to result in net negative impacts on abundance and/or performance under future global change scenarios.

### 4.1 Growth and net calcification

Few studies have assessed how coralline algae growth rates such as linear extension and changes in surface area will be impacted by climate change (but see: (Ragazzola et al. 2012, McCoy 2013, McCoy and Ragazzola 2014, Kolzenburg et al. 2021, Cornwall et al. 2020). This information is important to better understand how changes to physiology may alter the biology and ecology of coralline algae. For instance, McCoy and Ragazzola (2014) found that robust, vertically thick crustose species decreased in overall thickness but did not change their internal carbonate chemistry in response to acidification. Conversely, thin‐crusted species did not change their thickness but did change the density of internal carbonate material. Crustose coralline algal species exhibit both vertical and lateral growth, which may enable them to be more plastic in response to acidification, whereas articulated species, such as *Calliarthron tuberculosum,* primarily grow vertically. Here, we found reduced growth as both linear extension and surface area in *C. tuberculosum* in response to warming and combined warming and increased *p*CO_2_. Kolzenburg et al. (2021), however, found that the effects of warming, acidification, or combined warming and acidification on linear extension of the articulated coralline, *Corallina officinalis,* differed across populations. Climate change drivers had positive effects on growth in populations from the northern edge of *C. officinalis'* range, mixed effects (neutral, positive, or negative) in central populations, and negative effects in populations at the southern range edge. Importantly, changes in linear extension and surface area could alter the habitat available for species that rely on articulated corallines for shelter (McLean 1962). Given that the impacts of climate change stressors on growth differ across morphotypes and populations, it will be important for future work to focus on the mechanistic underpinnings of these differences as they have important implications for coralline persistence and ecosystem functioning.

For many calcified marine algae, calcification is necessary for growth and survival. Many studies have shown that coralline algae calcification rates are reduced in low pH and warming conditions. We found that the impacts of warming and combined warming and acidification had a greater impact on growth than acidification. Conversely, acidification and combined warming and acidification had a greater impact on calcification than warming. Together, these results suggest that at ambient temperature, *Calliarthron tuberculosum* may partially compensate for reductions in calcification due to increased *p*CO_2_ by reducing the proportion of carbonate in skeletal materials while still maintaining growth. These findings are similar to what has been shown for some crustose species (McCoy and Ragazzola 2014, Williams et al. 2021). This reduction in carbonate material, however, could have implications for structural integrity (Ragazzola et al. 2012) and susceptibility to grazing (Johnson and Carpenter 2012, McCoy and Kamenos 2018, Rich et al. 2018, Rasher et al. 2020). As storm frequency and intensity increase due to climate change, a reduction in standing kelp biomass may also reduce wave buffering capacity within kelp beds, where *C. tuberculosum* lives (Byrnes et al. 2011). Reduced skeletal density (and decreased structural integrity) coupled with increased physical disturbance could lead to reductions in articulated coralline biomass on rocky reefs and subsequently alter the competitive dynamics between corallines and other primary space occupiers.

Species inhabiting dynamic environmental conditions may be better prepared to deal with future climate change. We hypothesized that subtidal kelp forest ecosystems along the coast of California may be more resilient to future environmental change due to the natural variability they are currently exposed to. However, we found that both warming and acidification negatively impacted the growth and calcification of *Calliarthron tuberculosum* in our 28‐d experiment. Interestingly, Kram et al. (2015) did not detect an effect of acidification on the articulated intertidal coralline, *Jania adhaerens,* or the crustose coralline alga, *Lithothamnion californicum* which were both collected intertidally less than 3 km from our collection site. Kram et al. (2015) did find reductions in growth as a consequence of acidification on the articulated coralline, *Corallina vancouveriensis,* but no effects of warming either in isolation or in combination with acidification. We measured ranges of pH of 7.57–8.15 and temperature of 11.4–17.7°C over a 6‐month duration at our subtidal kelp forest site. Intertidal zones are known for extreme fluctuations in pH with a range of up to 0.8 pH units over a diel cycle (Chan et al. 2017). Kolzenburg et al. (2021) found that the effects of warming and acidification on growth and calcification in the intertidal coralline, *Corallina officinalis* were population–specific, such that local regional conditions impacted how species responded to future changes. Together, these results suggest that local adaptation/acclimation to environmental means and variability will play a role in mediating species responses to future changes and that corallines inhabiting these dynamic intertidal zones may be more acclimated/adapted or otherwise resilient to the levels of warming and acidification that are predicted to occur in the near‐term future due to their prior exposure to extreme variability.

### 4.2 Photophysiology

Changes in photophysiological performance in marine macrophytes can alter reef primary production. Researchers have suggested that the increase in CO_2_ due to climate change could be beneficial for marine algae directly due to an increase in this substrate for photosynthesis or indirectly if species are able to down‐regulate costly carbon‐concentrating mechanisms (CCMs; Koch et al. 2013). The fertilizing effect of CO_2_ on photosynthetic performance has been shown in marine macrophytes (Kroeker et al. 2013), though many also deviate from this general trend (Hofmann et al. 2012, Noisette et al. 2013, Kolzenburg et al. 2021). We were unable to detect an effect of increased CO_2_ on the photophysiology of *Calliarthron tuberculosum*. McCoy et al. (2020) also did not detect an effect of acidification on the photophysiology of the articulated coralline alga, *Ellisolandia elongata,* and suggest that this may be due, in part, to insufficient light energy during their experiment to fully take advantage of carbon fertilization (i.e., increased photosynthetic rates due to increased availability of CO_2_). Since light acclimation plays an important role in shaping species photosynthetic responses, experimental light conditions will influence how species respond to increased CO_2_ availability and their underlying photosynthesis‐irradiance curves (Egilsdottir et al. 2016). A light limitation may have played a role in our experiment as well since individuals were acclimated to light levels that were approximately 50% of those seen in the field during the same time of year. Importantly, the light levels in our experiment do occur naturally within subtidal California kelp forests (Gerard 1984), particularly when shaded by a thick surface or sub‐surface kelp canopy. Future work disentangling the interaction between irradiance and acidification will be important to more fully understand how acidification is likely to impact marine algae across seasons.

Under warming (both in isolation and combined with acidification), we saw decreased photosynthetic performance and decreased growth and calcification. The nonlinear effects of temperature on species' performance mean that warming has the potential to be either positive or negative, depending on where acclimation temperatures sit relative to the species' thermal performance curve (Brown et al. 2004). Our finding here is particularly interesting since during our experiment, incoming ambient seawater temperatures were approximately 2°C cooler than the average in situ temperature measured between October 2013 and April 2014. Therefore, our warming treatment was similar to the average at a different time of year. Importantly, it is the balance between multiple physiological rates (e.g., photosynthesis, respiration, and calcification) across seasons that will determine a species' net growth and persistence over time. In our study, the potential benefits of warming on photosynthetic performance did not outweigh the costs associated with changes to other metabolic processes.

### 4.3 Mineralogy

The number of studies investigating the effects of OA on the mineralogy of coralline red algae has increased in recent years due to concerns about the susceptibility of their high‐Mg calcite skeletons to acidification. Previous studies have assessed whether corallines are able to change the mol % Mg at reduced pH and saturation states, reporting that some species respond to acidification with reductions in the mol % Mg found within their carbonate skeletons (Ries 2011, Ragazzola et al. 2016), whereas other studies do not (Nash et al. 2015a). In articulated corallines, one study by Egilsdottir et al. (2013) found a positive relationship between Mg/Ca ratios and pH in new skeletal material deposited during their 3‐week study. Yet, similar to a study on the articulated coralline, *Jania rubens* (Porzio et al. 2018), our study on the articulated *Calliarthron tuberculosum* did not find significant effects of *p*CO_2_ or temperature on mineralogy. This variability in species mineralogical responses could be due to the magnitude of acidification used in experiments. Our experiment decreased pH in acidification treatments by ~0.50 units, which is similar in magnitude to Porzio et al. (2018), whereas the study by Egilsdottir et al. (2013) only reduced pH by ~0.30 units. It is possible that articulated corallines are able to utilize mineralogical plasticity under moderate acidification, but under extreme acidification, this plasticity is no longer possible. Furthermore, we found that within medullary cells, the effect of acidification on the mol % Mg was dependent on the temperature level. At ambient temperature, high *p*CO_2_ increased the Mg incorporation into cells, whereas at high temperature, high *p*CO_2_ decreased the mol % Mg. The temperature has been shown to be positively correlated with Mg incorporation in biominerals and high *p*CO2 leads to lower Mg content, potentially due to preferential Mg leaching or intentional reductions in Mg incorporation to reduce vulnerability to acidification (Pauly et al. 2015). Although we found a reduction in mol % Mg in medullary cells in response to combined warming and acidification, it is unclear why Mg incorporation would increase in response to high *p*CO_2_ at ambient temperatures. Future studies should focus on other biological and physiological processes that may interact with Mg incorporation, as well as improving experimental designs to detect tipping points and thresholds in species’ responses to OA and warming.

Techniques utilizing bulk tissue samples to measure mol % Mg (e.g. XRD) may mask important fine‐scale variability, which could be crucial to how these organisms persist in dynamic ecosystems, such as tide pools and upwelling regions. Our study found that mol % Mg is higher in *Calliarthron tuberculosum* medullary cells compared to cortical cells, irrespective of our experimental treatments. Medullary cells form the central core of filaments that run from geniculum to geniculum. These internal cells had on average, ~125% higher Mg/Ca ratios. The mechanism—or purpose—for this dramatic difference between different cell types remains unknown but may be due to biology or abiotic conditions. For instance, relationships between growth rate and Mg/Ca ratios have been found; however, these measurements are confounded by temperature. At elevated temperatures, Mg/Ca ratios in crustose coralline algae have been reported to increase, however, so do growth rates (Kamenos and Law 2010). It is possible that internal medullary cells (which are responsible for increases in linear extension) simply grow faster than cortical cells (which thicken the alga), which could explain why medullary cells have higher mol % Mg, if the growth rate is correlated with Mg incorporation (Nash and Adey 2018). Alternatively, the ratio of Mg/Ca in seawater has been shown to control Mg fractionation in coralline red algae (Ries 2006). Therefore, it is possible that the patterns seen here are due to differences in the ratios of Mg/Ca in the calcifying fluid of cortical vs. medullary cells. Ultimately, the reduced Mg incorporation in outer cortical cells may be a strategy to decrease solubility in cell types closer to the external environment. More studies are needed to determine how widespread this pattern is across cell types in articulated corallines and how these differences arise.

## 5 CONCLUSIONS

The present study found that *Calliarthron tuberculosum* growth and calcification were reduced under simulated acidification and warming predicted for the year 2100, relative to known ambient conditions. Reductions in net calcification and changes to growth could have consequences for both the abundance and integrity of *C. tuberculosum*. We observed variation in the mol % Mg within carbonate skeletons of different cell types. Future work to determine whether morphological or structural advantages result from this mineralogical variability will be important for predicting the emergent effects of global warming and OA on this species. The impacts of warming on photosynthetic rates and growth in *C. tuberculosum* also suggest that warming, not ocean acidification, may have a bigger impact on this alga's ability to maintain current levels of primary productivity, but acidification will certainly impact net calcification as well and thus ecological function. The changes to biology brought on by warming and acidification measured in this study could have cascading effects on species that rely on *C. tuberculosum* for food, habitat, and other important ecological processes such as settlement cues.

Our study was conducted on individuals collected from a single location and lasted only 28 d in duration. Future research should investigate the potential for acclimation over longer periods of time and or seasonal variability in responses to these stressors. Furthermore, variability in temperature and pH occurs spatially across the geographic range of *Calliarthron tuberculosum*. Local adaptation/acclimation may provide key insights into the mechanisms that coralline algae have evolved to persist in environments similar to those predicted to occur by the end of the century.

##  

We would like to thank Phil Zerofski for help in the field and Isabella Doohan, Molly Gleason, Emily Kelly, Susan Kram, Maggie Johnson, and Garrett Stewart for help in the laboratory. This research was supported by the CSU Council for Ocean Affairs, Science & Technology (COAST), the Dr. Earl and Ethyl Myers Oceanographic and Marine Biology Trust, NSF OCE RIG‐1420900 (to N.N.P.), and NOAA Grant #NA10OAR4170060 to the California Sea Grant College Program (Project #R/CC‐05 to J.E.S., S.L.H., and N.N.P), through NOAA's National Sea Grant College Program, U.S. Department of Commerce. The statements, findings, conclusions, and recommendations are those of the author(s) and do not necessarily reflect the views of California Sea Grant, state agencies, NOAA, or the U.S. Department of Commerce.

## AUTHOR CONTRIBUTIONS

**E.M. Donham:** Conceptualization (equal); data curation (equal); formal analysis (equal); investigation (equal); methodology (equal); visualization (lead); writing – original draft (lead); writing – review and editing (lead). **S. Hamilton:** Conceptualization (equal); data curation (supporting); formal analysis (supporting); funding acquisition (equal); investigation (equal); methodology (equal); project administration (equal); supervision (lead); writing – original draft (supporting); writing – review and editing (supporting). **I. Aiello:** Conceptualization (supporting); formal analysis (supporting); methodology (supporting); resources (supporting); supervision (supporting); writing – review and editing (supporting). **N. Price:** Conceptualization (equal); formal analysis (supporting); funding acquisition (equal); investigation (supporting); methodology (supporting); resources (supporting); supervision (supporting); writing – review and editing (supporting). **J. Smith:** Conceptualization (equal); funding acquisition (equal); methodology (equal); project administration (equal); resources (equal); supervision (supporting); writing – review and editing (supporting).
